# Infantile de novo primary antiphospholipid syndrome revealed by neonatal stroke

**DOI:** 10.1186/1546-0096-9-S1-P263

**Published:** 2011-09-14

**Authors:** E Merlin, E Doré, S Chabrier, A Marques Verdier, JL Stéphan

**Affiliations:** 1 CHU Clermont-Ferrand, INSERM CIC 501, 63003 Clermont-Ferrand, France; 2 CHU Saint-Etienne, Hôpital Nord, 42000 Saint-Etienne, France

## Ba

Antiphospholipid antibody syndrome (APS) is a rare condition in childhood. Some cases have been reported in neonates, and it is believed that most of them result from a transplacental transfer of antiphospholipid antibodies (APLA) from the mother to the foetus.

## Case

the first child of a 28 year healthy mother with no history of auto-immunity nor thrombotic events. The pregnancy was complicated by diabetes. The birth arose after a 39 weeks pregnancy, by normal vaginal delivery. The male newborn weighted 3830 g (P90). The clinical examination was normal. The third day, he exhibited clonic seizure of the right hemi-body. EEG demonstrated left temporal spikes. Cerebral ultrasonography and MRI showed infarction in the territory of the left middle cerebral artery. Prothrombin time and activated partial thromboplastin time, antithrombin III, protein C, protein S and homocystine levels were normal. There was no mutation of the factor II or V. Serology for antiphospholipid antibodies and detection for antiβ2gp1 were negative in the child and the mother serum. One year later, a new systematic screening showed a high titer of anticardiolipid antibodies (Table 1). Antinuclear antibodies were negative. None of those antibodies were found in the maternal serum. All those features persisted on a second testing 12 weeks later.

**Table 1:** Biological features of the child and the mother

			Time after birth			
			
			5 days		1 year	
			
		*Normal values*	Child	Mother	Child	Mother
**KCT (sec)**			43/34		52/35	36/36
**Anticardiolipid AB**	IgM (UMPL/ml)	*<10*	<5	<1	4	<1
	IgG (UGPL/ml)	<*11*	<5	<1	191	<1
**Anti β2gpI AB**	IgM	<*10*	<5	<1	<1	<1
	IgG	<*20*	<5	<1	258	<1
**VDRL**			-	-	NEG	NEG
**TPHA**			-		NEG	NEG

## Conclusion

Contrary to what is usually thought, neonatal APS not always result from the transplacental transfer of APLA. Our case highlights the importance of considering the maternal status when reporting on neonatal APS; and of considering the possibly of APS even in the absence of antibodies in the mother.

